# Discoveries Interview: Professor Harold F. Dvorak on the discovery of Vascular Endothelial Growth Factor (VEGF)

**DOI:** 10.15190/d.2016.9

**Published:** 2016-07-07

**Keywords:** VEGF discovery, VPF, angiogenesis, pioneer, groundbreaking, cancer angiogenesis

**Figure 1:**
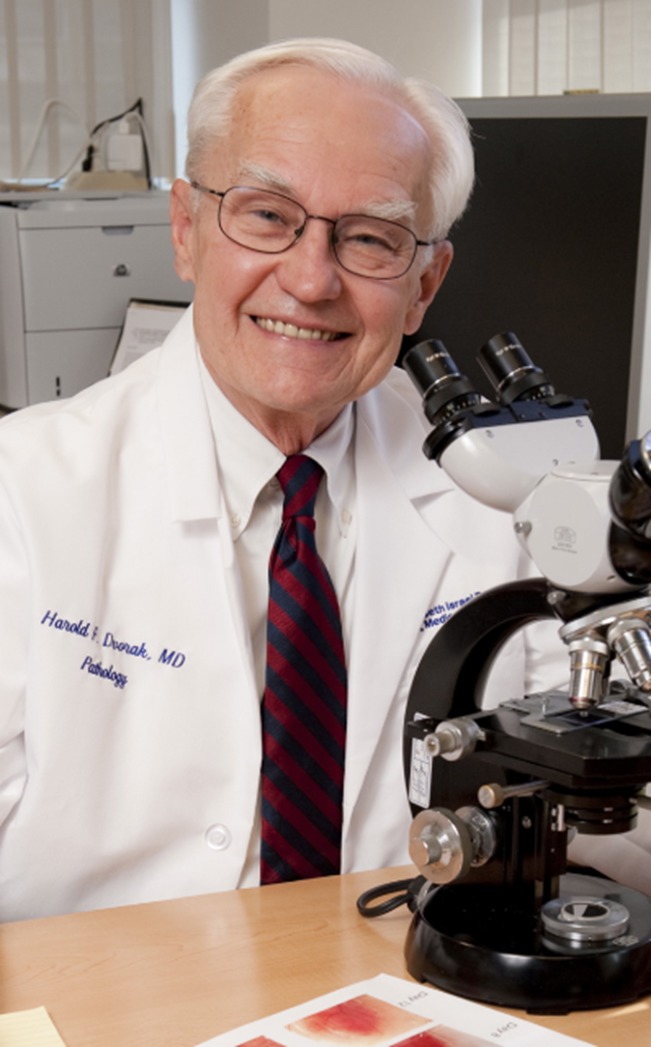
Professor Harold F. Dvorak

**Dr. Harold F. Dvorak **is the founding Director of the Center for Vascular Biology Research (CVBR) at the Beth Israel Deaconess Medical Center (BIDMC) and the Distinguished Mallinckrodt Professor of Pathology at Harvard Medical School and BIDMC. Professor Dvorak did his residency training in pathology at Massachussetts General Hospital (MGH), followed by two years of post-doctoral fellowship at NIH and 16 years as Staff Pathologist at MGH. Prof. Dvorak was for 26 years the Chair of the Department of Pathology at BIH and then BIDMC. 

Prof. Dvorak is well known for the **discovery of Vascular Endothelial Growth Factor (VEGF),** receiving the Canada Gairdner International Award in 2014. Prof. Dvorak received numerous other prestigious **awards**, including, but not limited to: *Rous-Whipple Award* (American Society for Investigative Pathology), *Lefoulon-Delalande Grand Prix* (French Academy of Sciences (with Judah Folkman and Napoleone Ferrara)), *Szent-Gyorgyi Prize* for progress in cancer research (discovery of VPF), *Earl P. Benditt Award* (North American Vascular Biology Organization), *Distinguished Service Award* (Association of Pathology Chairs), *American Society for Investigative Pathology Gold-Headed Cane Award*. 

Prof. Dvorak is also an **elected fellow** of American Association for the Advancement of Science, National Foundation for Cancer Research and Association of American Physicians.

“In 1983, Dr. Dvorak and his colleagues were the first to demonstrate that tumor cells secreted vascular endothelial growth factor (VEGF), known at the time as vascular permeability factor or VPF. This seminal discovery provided the molecular basis for the field of angiogenesis.” (Canada Gairdner International Award, 2014)^1^

## 1. What is VEGF-A and why is it important

VEGF-A is a highly conserved, disulfide-bonded dimeric glycoprotein of Mr ~45 kD. It shares low but significant sequence homology with platelet-derived growth factor (PDGF), and, like PDGF, has cysteines that form integral inter- and intra-chain bonds. VEGF-A is the founding member of the VPF/VEGF family of proteins that also includes VEGFs B, C and D as well as PlGF (placenta growth factor) and a related viral protein, VEGF-E. VEGF-A has critical roles in vasculogenesis, tumor and other examples of pathological angiogenesis (e.g., wound healing, chronic inflammation), and lymphangiogenesis, acting through receptors (VEGFR-1, VEGFR-2 and neuropilin) that are expressed on vascular endothelium as well as on certain other cell types^2-5^. The product of a single gene, VEGF-A is alternatively spliced to form several proteins of different lengths, properties and functions. VEGF-A was originally discovered as a vascular permeabilizing factor (VPF) with a potency some 50,000 times that of histamine^6,7^. It is also an endothelial cell motogen and mitogen, profoundly alters the pattern of endothelial cell gene expression, and protects endothelial cells from apoptosis and senescence^3^. Recently, VEGF-A has been found to have additional critical roles in hematopoiesis and in development and maintenance of the nervous system. 

VEGF-A is overexpressed in human and animal carcinomas and in healing wounds (e.g., in skin wounds, myocardial infarcts and strokes), in chronic inflammation (e.g., delayed hypersensitivity, rheumatoid arthritis, psoriasis), and in various retinopathies^3,5^. The increased microvascular permeability induced by VEGF-A leads to tissue edema and stroma formation, a characteristic of all these entities^3^. Extravascular fluid accumulation is particularly prominent in tumors growing in body cavities such as the peritoneum (ascites tumors). Extravasated plasma fibrinogen undergoes clotting to fibrin, providing a provisional stroma for endothelial cell and fibroblast migration and generation of mature stroma (desmoplasia in the case of tumors, scar formation in wound healing)^3^. The similarities between tumor growth and wound healing suggest that in important respects “tumors are wounds that do not heal”^5^.

VEGF has been most important in the clinic as a therapeutic target. The concept of anti-angiogenesis as an approach to tumor therapy has a long history and was brilliantly formulated by the late Judah Folkman^8^. Hopes for this approach were encouraged by the success that anti-VEGF-A antibodies, as well as drugs targeting VEGF-A or its receptors (VEGFR), had on inhibiting the growth of many rodent tumors. Anti-VEGF/VEGF receptor drugs are currently used in the treatment of many tumors and in wet macular degeneration where they have provided important clinical benefit (reviewed in^5^).

## 2. How did you discover VEGF and how our knowledge about it and its involvement in angiogenesis evolved over time?**

In the mid-1970s I found that the stroma of solid tumors contained fibrin, a protein that results from the clotting of the plasma protein fibrinogen^6^. I reasoned that, for fibrin to be deposited in tumor stroma, tumor blood vessels had to be leaky to plasma fibrinogen. I therefore postulated the existence of a tumor-secreted vascular permeability factor (VPF) that was responsible for the characteristic hyperpermeability of tumor blood vessels. We found that cultured tumor cells secreted a VPF and characterized it to be a protein whose activity was not inhibited by anti-histamines and other classic inhibitors of vascular permeability^6^. Therefore, we concluded that our VPF acted directly on blood vessel endothelium. Donald Senger and I then purified VPF to homogeneity, determined its N-terminal sequence, and raised the first antibody against it^7^. This antibody blocked peritoneal accumulation of fluid in animals bearing ascites tumors, providing strong evidence that VPF was the agent responsible for the accumulation of fluid in body cavities induced by tumors. Subsequently, we developed the first immunoassay to assess VPF and demonstrated VPF to be abundantly present in a wide variety of animal and human tumors and tumor ascites fluids. Subsequently, two groups (Connolly at Monsanto, Ferrara at Genentech) cloned VPF and renamed it VEGF because of its ability to stimulate proliferation of cultured endothelial cells.

## 3. How our knowledge on this discovery will help understand and target human disease? 

Treatments that reduce VEGF-A expression or inhibit its action are now used in the clinic as an anti-angiogenesis approach to cancer therapy. Antibodies that neutralize VEGF-A strikingly inhibit tumor growth in mice and, along with receptor tyrosine kinase inhibitors, have had some success in prolonging the life of patients with colon, kidney and some other types of cancer. Antibodies have been extremely beneficial in the treatment of the leaky, angiogenic blood vessels characteristic of wet macular degeneration, reversing loss of eye sight.

## 4. What advice do you have for young scientist?

Think big, outside the box, be willing to take career risks to solve the most important problems, don’t waste your time on the small stuff, work hard, be totally honest and rigorous in your work, no cheating. 

### 5. In your opinion, what are the most challenging, promising and/or the most rewarding areas of research?

The demographics of society throughout the developed world are changing rapidly. There is an ever-increasing population of elderly who are living longer and who are plagued by chronic diseases such as cancer, heart disease and neurodegenerative illnesses. Of these, the last is least studied and in greatest need of research.
